# Microbial intestinal therapeutics: An annotated selection of World Wide Web sites relevant to the topics in microbial biotechnology

**DOI:** 10.1111/1751-7915.13819

**Published:** 2021-04-30

**Authors:** Lawrence P. Wackett

**Affiliations:** ^1^ Department of Biochemistry, Molecular Biology and Biophysics, BioTechnology Institute University of Minnesota St. Paul MN 55108 USA

## Bacterial therapeutic for phenylketonuria (PKU)

https://www.nature.com/articles/nbt.4222

Phenylketonuria, often known as PKU, is a genetic disorder resulting incomplete metabolism of phenylalanine, and it causes extreme neurotoxicity if not strictly controlled by diet, which is difficult. In this study an *Escherichia coli* strain engineered to degrade phenylalanine to harmless products was tested in a mouse model.

## Genetically modified bacteria treating illness

https://www.npr.org/sections/health‐shots/2019/03/08/687370312/a‐gulp‐of‐genetically‐modified‐bacteria‐might‐someday‐treat‐a‐range‐of‐illnesses

This news article discusses microbial disease therapies with a focus on treating PKU as an example.

## Therapeutic microbes to tackle disease

https://www.nature.com/articles/d41586‐020‐00201‐6

This review discusses single engineered bacteria strains and microbial consortia used in treating various human diseases.

## Microbial therapies for inflammatory bowel disease

https://www.ncbi.nlm.nih.gov/pmc/articles/PMC6335320/

Inflammatory bowel disease is a complex set of disorders that involve intestinal microbes and host inflammatory responses. Human studies are suggesting some promising lines for developing new therapies.

## Gut microbiota modulation for colorectal cancer

https://www.nature.com/articles/s41388‐020‐1341‐1

This review article discusses research on colonization resistance for pathogenic bacteria, mucosal immunomodulation and probiotic use in prevention and treatment of colorectal cancer.

## Can intestinal microbes help fight cancer?

https://www.sciencemag.org/features/2020/11/can‐patients‐gut‐microbes‐help‐fight‐cancer

This review article focuses on the interactions between microbes and host T cells in the intestine.

## Gut microbes and ulcerative colitis

https://www.medicalnewstoday.com/articles/gut‐microbes‐could‐be‐key‐to‐treating‐ulcerative‐colitis

This article summarizes findings that some people with ulcerative colitis lack a specific bacterium involved in bile acid metabolism and it considered supplementation with the missing microbe.

## Q&A for fecal transplants

https://newsnetwork.mayoclinic.org/discussion/mayo‐clinic‐q‐and‐a‐fecal‐transplant‐for‐treatment‐of‐clostridium‐difficile/

This question and answer page from the Mayo Clinic covers fecal transplants for the treatment of *Clostridium difficile*.

## Microbial cocktails for *C. diff*.

https://www.nature.com/articles/s41587‐020‐00765‐8

While undefined fecal transplants have proven to be effective therapeutically, this article describes an effort to produce a capsule of bacteria with a more defined composition to be used to treat *C*. *difficile*.

## *Clostridium difficile*

http://www.antimicrobe.org/new/b113.asp

This article discusses all of the standard treatments for *C*. *difficile*, from antibiotics to biotherapies.

## Microbiome‐based therapies

https://www.biospace.com/article/the‐brave‐new‐world‐of‐microbiome‐based‐therapies/

This article focuses on various business startups and partnerships that have emerged in an effort to commercialize microbe‐based disease therapies.

## The “psychobiome”

https://www.sciencemag.org/news/2020/05/meet‐psychobiome‐gut‐bacteria‐may‐alter‐how‐you‐think‐feel‐and‐act

A relatively recent area of gut microbiome research focus on how microbes might help treat ailments such depression and insomnia. This general article discusses developments in this field.

